# Incorporation of Nonmetal Group Dopants into g-C_3_N_4_ Framework for Highly Improved Photocatalytic H_2_ Production

**DOI:** 10.3390/nano11061480

**Published:** 2021-06-03

**Authors:** Weinan Xing, Ke Cheng, Yichi Zhang, Jie Ran, Guangyu Wu

**Affiliations:** 1 Co-Innovation Center for the Sustainable Forestry in Southern China, College of Biology and the Environment, Nanjing Forestry University, Nanjing 210037, China; kecheng1221@126.com (K.C.); yichizhang2021@163.com (Y.Z.); hyiyi1232021@163.com (J.R.); 2 Key Laboratory of Advanced Energy Materials Chemistry (Ministry of Education), College of Chemistry, Nankai University, Tianjin 300071, China; 3 Key Laboratory of Functional Molecular Solids, Ministry of Education, Anhui Normal University, Wuhu 241000, China; 4 Jiangsu Provincial Key Laboratory of Palygorskite Science and Applied Technology, Huaiyin Institute of Technology, Huaian 223003, China

**Keywords:** g-C_3_N_4_, nonmetal group dopants, photocatalytic hydrogen production, semiconductors, energy storage and conversion

## Abstract

The incorporation of nonmetal group dopants into a graphitic carbon nitride (g-C_3_N_4_) framework is fabricated by adding a small amount of hexamethylenetetramine during the thermal polymerization process. The material shows an excellent visible-light photocatalytic H_2_ production performance that is eight times higher than bulk g-C_3_N_4_. This outstanding performance is ascribed to the introducing of N-doped carbon, which not only enhances the light absorption and favorsa narrower band gap, but also upshifts the conductionband (CB) potential, resulting in a better reduction ability of electrons. This discovery has potential significancefor the designing of high performance, economic, and environmental friendly photocatalyst for solar energy conversion.

## 1. Introduction

Environmental deterioration and the global energy crisis are both increasingly serious problems. Photocatalytic water splitting can be used to obtain hydrogen (H_2_), which is supposed to replace fossil fuel for future energy demand [1,2]. Among semiconductor catalysts, graphitic carbon nitride (g-C_3_N_4_) shows enormous superiorities such as: low-cost starting material, visible light response, simple synthesis method, suitable band position (about 2.7 eV), and chemical stability [3]. Moreover, bulk g-C_3_N_4_ still suffers from its high recombination efficiency of photogenerated electrons and holes and insufficient optical absorption, which lead to unsatisfactory photocatalytic activity. To date, various strategies have been explored to improve the photocatalytic activity; for example, electronic structure regulation [4,5,6], nanostructure design [7,8,9], crystal-structure engineering [10], and heterostructure construction [11,12,13,14].

The electronic structure regulation design of semiconductors plays a vital function in optimizing photocatalytic efficiency [15,16]. A series of metal or nonmetal dopants have been used to achieve a high photocatalytic performance. Tu et al. [17] constructed tunable nitrogen vacancies in a g-C_3_N_4_ nanosheet photocatalyst, which improved light harvesting and carrier separation, and it was thus revealed as an efficient photocatalysts for H_2_ evolution. Zhu et al. [18] prepared a Ce-doped biomass carbon-based g-C_3_N_4_ photocatalyst, which exhibited high photocatalytic performance for 2-mercaptobenzothiazole degrading performance. Song et al. [19] fabricated C and O co-doped carbon nitride photocatalyst, which extended the optical absorption range and narrowed down the bandgap, thus displaying a better photocatalytic CO_2_ reduction performance. The above samples obviously demonstrate that element-doped g-C_3_N_4_ is a good and useful way to raise light absorption and improve the photocatalytic activity of g-C_3_N_4_. Nevertheless, compared with simple element doping, a functional group such as the dopant would produce more reserve forces in optimizing the light absorption and enhancing the catalytic efficiency.

Herein, a facile and reliable strategy is developed to prepare nonmetal group doped g-C_3_N_4_ through thermal polymerization by adding hexamethylenetetramine. Hexamethylenetetramine has a similar elementary composition to melamine. After the thermal polymerization, the hexamethylenetetramine forms N-doped carbon, whichis incorporated into the g-C_3_N_4_ framework. As a result, the as-prepared sample shows eight times higher photocatalytic activity of H_2_ evolution than bulk g-C_3_N_4_. As a result, we now have an insight into the influence factors of this unique performance by light absorption and fluorescence characterizations, among other things. 

## 2. Materials and Methods

### 2.1. Chemicals and Reagents

Melamine, hexamethylenetetramine, and triethanolamine (TEOA, analytical reagent grade) were purchased from Aladdin (Shanghai, China) and were used without further purification. 

### 2.2. Synthesis of Bulk g-C_3_N_4_Photocatalysts

Bulk g-C_3_N_4_ was synthesized by the thermal polycondensation method. Briefly, 2.0 g of melamine was put into an alumina crucible (20 mL) with a cover, then the alumina crucible was heated to 550 °C for 2 h at a heating rate of 2 °C min^−1^ in a muffle furnace. Finally, the sample was collected and ground into powder, denoted as CN. 

### 2.3. Synthesis of Nonmetal Group-Doped g-C_3_N_4_Photocatalyst

The nonmetal group-doped g-C_3_N_4_ sample was prepared as follows: CN (500 mg) was mixed with hexamethylenetetramine (50 mg) in 30 mL deionized water. The suspension was stirred at 80 °C in a water bath until the water had been removed completely. Then, the obtained solid mixture was put into a porcelain boat. Finally, the nonmetal group-doped g-C_3_N_4_photocatalyst was obtained by pyrolysising the precursor at different temperatures (500 °C, 550 °C, and 600 °C) for 2 h at a ramping rate of 5 °C min^−1^. The obtained samples were denoted as CN-1, CN-2, and CN-3. 

### 2.4. Characterization

X-ray diffraction (XRD) patterns were obtained on a RigakuD/max-2000 X-ray diffractometer equipped with Cu-Kα radiation. Fourier transform infrared (FTIR) spectra were measured with an IR Affinity-1 spectrometer in the range of 500–4000 cm^−1^, using KBr pellets. X-ray photoelectron spectroscopy (XPS) analysis was recorded on an American electronics physical HI5700ESCA system with X-ray photoelectron spectroscope using Al K (1486.6 eV) monochromatic X-ray radiation. The UV-vis diffuse reflectance spectra (DRS) were tested on a UV-vis spectrophotometer (PG, UH-4150) at room temperature. Scanning electron microscopy (SEM) images were observed on an FEI QUANTA200F. The N_2_ adsorption–desorption isotherms weremeasured by an AUTOSORB-1-MP surface analyzer at 77 K. The photoluminescence (PL) spectra were carried out on a FLUOROMAX-4C-TCSPC at room temperature.

### 2.5. Photocatalytic Test

The photocatalytic hydrogen evolution reactions were tested in asystem using a 300 W Xe lamp with a cut-off filter (λ > 400 nm). During the test, 100 mg of photocatalyst and 300 mL of aqueous solution were introduced into the quartz glass reactor. Meanwhile, 10 vol% of triethanolamine (TEOA) and H_2_PtCl_6_•6H_2_O were also used as a sacrificial agent and co-catalyst. The amount of H_2_ evolved was analyzed by on-line gas chromatography (Agilent 7890) with a thermal conductivity detector (TCD), and Ar was used as the carrier gas.

## 3. Results and Discussion

### 3.1. Composition and Structure of the as-Prepared Photocatalysts

**Figure 1:**
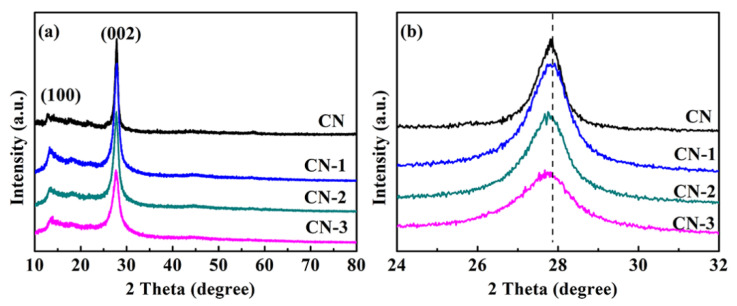
(**a**) XRD patterns and (**b**) the enlarged (002) peak of the CN, CN-1, CN-2, and CN-3 samples.

The crystal structures of the samples were confirmedby XRD patterns. As shown in Figure 1a, all the samples exhibit two characteristic diffraction peaks at 13.2° and 27.7°. The peak at 13.2° (100) is attributed to the in-planar repeating motifs of triazine units. The diffraction peak at 27.7° (002) is from the reflection of the graphitic structure [20,21]. The similar characteristic peaks indicate that the original g-C_3_N_4_ phase is well retained after the incorporation of nonmetal group dopants. In Figure 1b, the enlarged view of the (002) peak for the CN- CN-3 materials reveals that the peaks shift from 27.8° to 27.7°. According to Bragg’s law, the decrease invalue suggests an increase of interlayer distance. Such a slight shift of the (002) peak is expected because the incorporation of the nonmetal group is doped into the g-C_3_N_4_ framework, thus making the lattice spacing larger. The XRD results indicate that the nonmetal group has been incorporated into the g-C_3_N_4_ framework.

**Figure 2:**
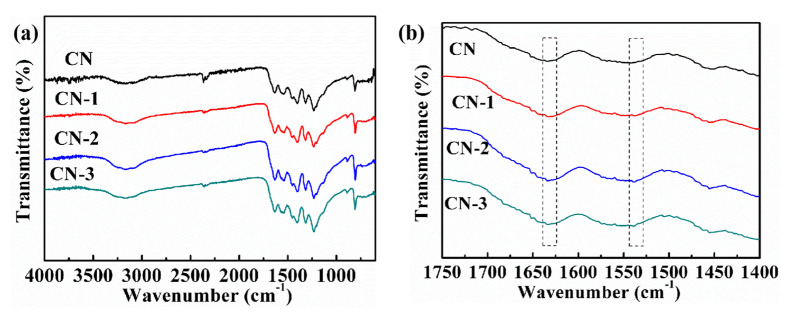
(**a**) FTIR spectra and (**b**) the enlarged FTIR spectra of the CN, CN-1, CN-2, and CN-3 samples.

The molecular structure information of the as-prepared photocatalyst are obtained by using FTIR spectroscopy. Similar FTIR spectra regarding the typical CN heterocycle skeletal vibration are revealed for the CN, CN-1, CN-2, and CN-3 samples (Figure 2a). The broad absorption bands at 3200–3400 cm^−1^ stem from the surface-bonded H_2_O molecules. The sharper peak at 808 cm^−1^ is related to the s-triazine ring, and several strong absorption bands in the region from 1200 to 1650 cm^−1^ belong to the typical stretching vibrations of carbon nitride aromatic heterocycles. It is worth noting that the two extra peaks at 1545 and 1630 cm^−1^ could be observed with increasing pyrolysis temperature (Figure 2b), indicating the existence of a C=C skeletal vibration band of aromatic domains [22]. This may be attributed to the additional carbon group incorporating into the framework of CN. In addition, the elemental analyses are used to explore the percentage of C and N in the photocatalyst. The results are exhibited in Table S1. The C/N ratio of CN is 0.76, which is similar to the theoretical value. After the incorporation of nonmetal group dopants, the percentage of C increases obviously, indicating that the hexamethylenetetramine has been decomposed and has formed the carbon species. This result is consistent with the FTIR analysis. Meanwhile, the C/N ratioincreases with the heat treatment temperature, suggesting the better condensation of the tri-s-triazine structure for the nonmetal group dopant-modified CN photocatalyst.

### 3.2. Chemical Composition and Surface States of the as-Prepared Photocatalysts

**Figure 3:**
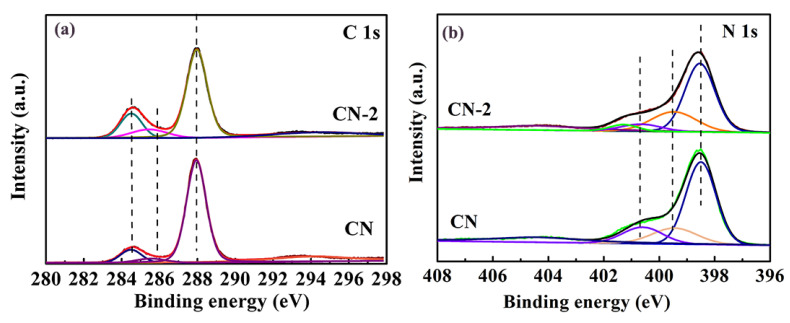
XPS spectra of CN and CN-2: (**a**) C 1 s and (**b**) N 1 s.

The elemental compositions and the surface chemical states of the as-prepared catalyst were studied by XPS measurement. Three sharp peaks are observed from the XPS survey spectra, which are assigned to C 1s, N 1s, and O 1s, respectively. (Figure S1). The existence of the O element may arise from the surface adsorption of water or O_2_. The XPS spectra of C (Figure 3a) can be fitted into three peaks centered at 287.9 eV, 286.0 eV, and 284.6 eV. The main high-resolution C 1 s spectra confirm the existence of sp^2^-hybridized carbon in the N containing aromatic ring (N–C=N,) and sp^2^ C atoms in the aromatic ring attached to the -NH_2_ group C-C bond, respectively [23]. The N 1s spectrum of CN (Figure 3b) can be resolved into three different peaks with binding energy at 400.6, 399.4 and 398.4 eV, which are attributed to N atoms bonded with H atoms, the tertiary nitrogen N-(C)_3_ groups, and sp^2^ bonded N in the triazine rings, respectively [24]. However, for the CN-2 sample, a new peak is located at 401.2 eV, which arises from the graphitic N-.The result suggest that the nonmetal group dopant is N-doped graphitic carbon. From the above XRD, FTIR, and XPS characterizations, we may conclude that the N-doped carbon modified g-C_3_N_4_ photocatalyst has been successfully prepared.

### 3.3. Optical Property of the as-Prepared Photocatalysts

**Figure 4:**
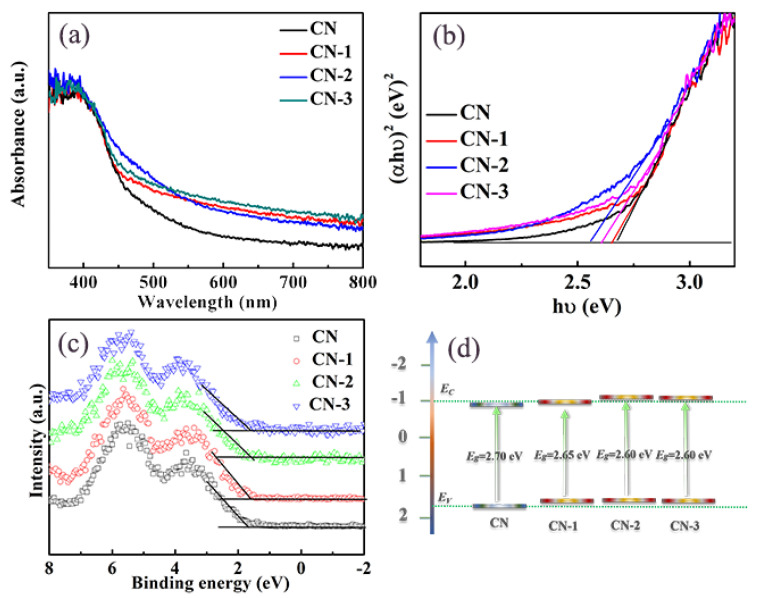
(**a**) UV-vis diffuse reflectance spectra, (**b**) (αhν)^2^ versus hν plot, (**c**) XPS valence, band spectra, and (**d**) Schematic band structure evolution of different samples.

Typically, the introduction of carbon species into the material would influence its light absorption properties directly. In order to investigate the effect of N-doped carbon modified g-C_3_N_4_ for light absorption, UV-vis diffuse reflectance spectroscopy (DRS) is employed. As shown in Figure 4a, the CN exhibits a conventional absorptionat about 475 nm, which is consistent with the reported value [25]. After the introduction of N-doped graphitic carbon, the UV-vis adsorptions edge of CN-1, CN-2, and CN-3 exhibit obvious red shifts compared to CN. This may be due to the N-doped carbon being incorporated into the g-C_3_N_4_ network; the lone pair electron from the graphitic N would trigger p-electron delocalization [26], which contributes to its enhanced photoabsorption. Meanwhile, the light absorption intensities of nonmetal group dopant-modified CN photocatalyst is significantly enhanced and is accorded with a color change from yellow to dark brown (Figure S2). Generally, the red shift of the adsorption edge often indicates a band gap narrowing of the material. Thus, the transformed Kubelka–Munk function of the energy of the light absorption edges is used to determine the band gap. As shown in Figure 4b, the intrinsic absorption edge of CN is 2.70 eV. After being modified with N-doped carbon, the band gaps for CN-2 are established as being 2.60 eV, which indicates the reduction of the band gaps. The reduced band gap energy is beneficial for electrons on the valence band (VB) to excite to produce photogenerated charge carriers. The reduced band gap energy and improved light absorption intensity are both better for facilitating photocatalytic H_2_ evolution.

From the UV-vis DRS results, the modification of nonmetal group dopants results in decreased band gap energy. The relative positions of the conductionband (CB) and valence band (VB) edges are a vital parameter of the photocatalytic reaction. In order to determine the positions of the CB andVB, the XPS VB spectroscopy are measured. From Figure 4c, the VB of CN, CN-1, CN-2, and CN-3 are estimated to be 1.75, 1.62, 1.50 and 1.55 eV, respectively. Through the band gap energy calculation, the CB positions are calculated to be −0.95 and −1.1 eV for CN and CN-2, respectively. The band structure of the as-prepared photocatalysts can also be obtained (Figure 4d). The CB of CN-2 shifts up to 0.15 eV, compared to CN. The negative shifting of CB is advantageous for the reduction reactions of electrons and is helpful for the photocatalytic H_2_ evolution. 

**Figure 5:**
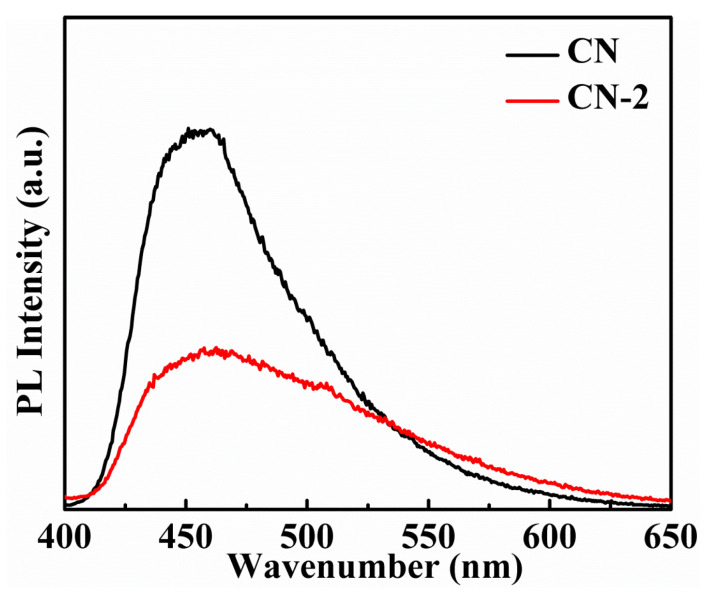
PL spectra of CN and CN-2 (325 nm excitation).

To explore the transmission behavior of photogenerated charge carriers after the introduction of N-doped carbon, the photoluminescence (PL) emission spectra are shown in Figure 5. The main emission peak of pure CN is around 460 nm and exhibits strong PL emission peaks. It has been reported that the intensity of PL emission spectroscopy reflects the recombination degree of charge carriers [27]. Thus, the strong PL emission peaks of CN indicate the high recombination efficiency of charge carriers. However, the intensity of the emission peak for CN-2 decreases sharply, suggesting that the N-doped carbon introduced into the g-C_3_N_4_ framework can drastically suppress the recombination of charge carriers. This is due to the incorporation of N-doped carbon into the CN network, which extends the π-conjugated system and effectively accelerates the transfer of charge carriers. Thus, a high efficiency of photocatalytic H_2_ production would be obtained. 

### 3.4. Photocatalytic Activity Investigation of the as-Prepared Photocatalysts

**Figure 6:**
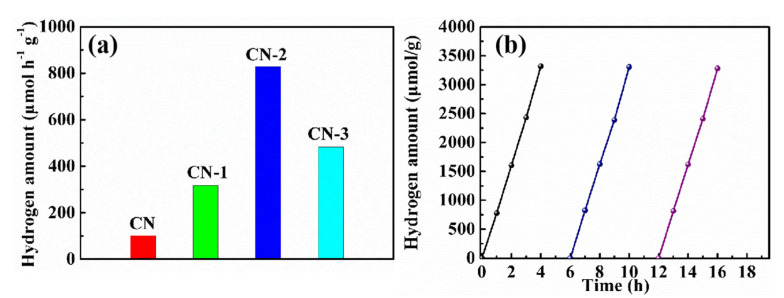
(**a**) Photocatalytic hydrogen evolution of CN, CN-1, CN-2, and CN-3 samples, (**b**) Stability test of hydrogen evolution for CN-2 under visible light irradiation.

The photocatalytic performance of the samples is evaluated by visible light-induced H_2_ evolution. (Figure 6). The CN photocatalyst shows a low photocatalytic H_2_ activity, with 101 μmol h^−1^g^−1^. This is because of its low light utilization and high recombination of the charge carrier. After the incorporation of N-doped carbon, photocatalytic H_2_ activity evidently increased. Notably, CN-2 (Figure 6a) shows the highest H_2_ production rate of 830 μmol h^−1^g^−1^, which is an eight-times-higher H_2_-production rate than CN. The photocatalytic H_2_ production rate is significantly better than that reported previously (Table S3).The improvement of the H_2_ production is attributed to the introduction of N-doped carbon species, which facilitate light harvesting, optimizing the band gap of g-C_3_N_4_ and resulting in a narrower bandgap and negative-shifted conduction band position. Meanwhile, the incorporation of nonmetal group dopants into the g-C_3_N_4_ framework is better for the separation and migration efficiency of the photo-induced charge carriers. In addition, the N_2_ adsorption–desorption isotherms and SEM images are used to explore the effect of morphology and specific surface area; the g-C_3_N_4_ presents a dense and stacked morphology characteristic (Figure S3). After the incorporation of nonmetal group dopants, the surface becomes loose and porous (Figures S4–S6). The specific surface area of CN-2is 22.74 m^2^g^−1^, which is higher than that ofg-C_3_N_4_(5.26 m^2^g^−1^) (Table S2). The higher surface area and porous structure are another reason for the increased photocatalytic activity. Considering the stability and reusability of the photocatalyst, recycling experiments are carried out. The H_2_-production stability confirms that the CN-2 sample can continue to work for 16 h without noticeable deactivation (Figure 6b), which indicates excellent stability for H_2_ evolution.

## 4. Conclusions

In summary, N-doped carbon modified g-C_3_N_4_ has been successfully prepared by introducing a slight amount of hexamethylenetetramine into the precursor. The optimal CN-2 photocatalyst shows that the photocatalytic H_2_ evolution rate is 830 μmol h^−1^g^−1^, which is over eight times higher than the bulk g-C_3_N_4_. The superior performance arises from introducing N-doped carbon into the network of g-C_3_N_4_. This readily tunes the band gap and improves the light absorption and separation efficiency of the charge carriers. This discovery could offer a new design idea for the fabrication of a highly efficient, low-cost and simple process material for the enlarged application of light utilization.

## Data Availability

The data presented in this study are available on request from the corresponding author.

## Supplementary Materials

The following are available online at https://www.mdpi.com/article/10.3390/nano11061480/s1, Figure S1, XPS survey spectra of CN and CN-2; Figure S2, Digital images of CN, CN-1, CN-2, and CN-3; Figure S3, SEM image of CN; Figure S4, SEM image of CN-1; Figure S5, SEM image of CN-2; Figure S6, SEM image of CN-3; Table S1, Elemental analysis of C and N content (wt.%) in CN, CN-1, CN-2, and CN-3; Table S2, BET surface area and H2 production rate of CN and CN-2 samples; Table S3, Hydrogen evolution of CN-2 and comparison with other reported g-C_3_N_4_ photocatalyst.

## Sample Availability

Samples of the compounds are not available from the authors.
