# Supplementary material for: Incorporation of Nonmetal Group Dopants into g-C3N4 Framework for Highly Improved Photocatalytic H2 Production
Source: Nanomaterials (Basel). 2021 Jun 3;11(6):1480. doi: 10.3390/nano11061480 (PMC8228239; doi:10.3390/nano11061480)
Supplement: Supplementary file 1 [file nanomaterials-11-01480-s001.zip › nanomaterials-1210617-SI.pdf]

nanomaterials

Incorporation of Nonmetal Group Dopants into g-C_3N_4 Framework for Highly Improved Photocatalytic H_2 Production

Weinan Xing [1,2,3,*], Ke Cheng [1], Yichi Zhang [1], Jie Ran [1] and Guangyu Wu [1,3,4,*]

[1] College of Biology and the Environment, Co-Innovation Center for the Sustainable Forestry in Southern China, Nanjing Forestry University, Nanjing 210037, China; yichizhang2021@163.com (Y.Z.); hyiyi1232021@163.com (J.R.); email
[2] Key Laboratory of Advanced Energy Materials Chemistry (Ministry of Education), College of Chemistry, Nankai University, Tianjin 300071, China
[3] Key Laboratory of Functional Molecular Solids, Ministry of Education, Anhui Normal University, Wuhu 241000, China
[4] Jiangsu Provincial Key Laboratory of Palygorskite Science and Applied Technology, Huaiyin Institute of Technology, Huaian 223003, China
* Correspondence: xingwn@njfu.edu.cn (W.X.); gywuchem@ njfu.edu.cn (G.W.)

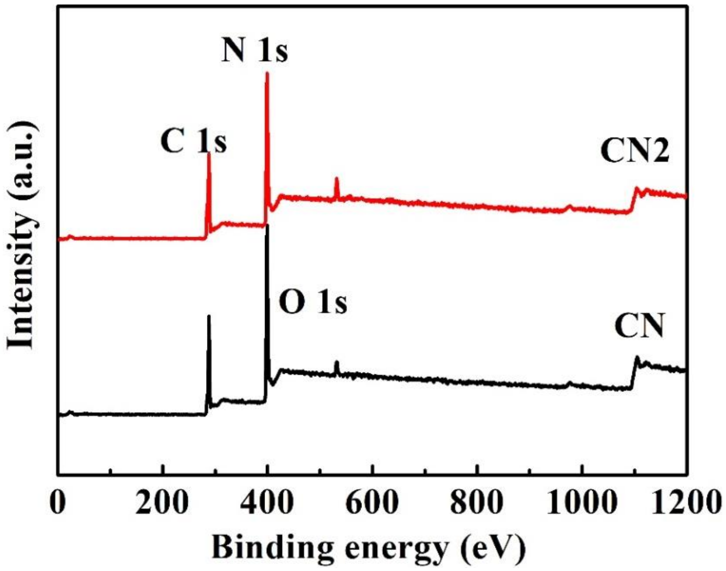

Figure S1. XPS survey spectra of CNand CN-2.

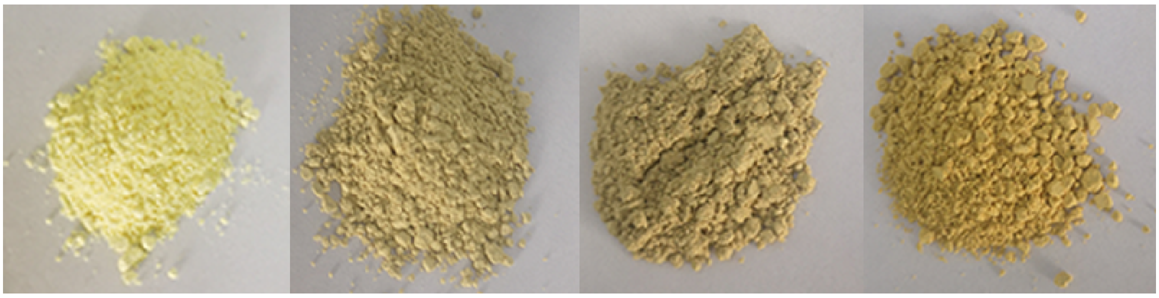

Figure S2. Digital images of CN, CN-1, CN-2 and CN -3.

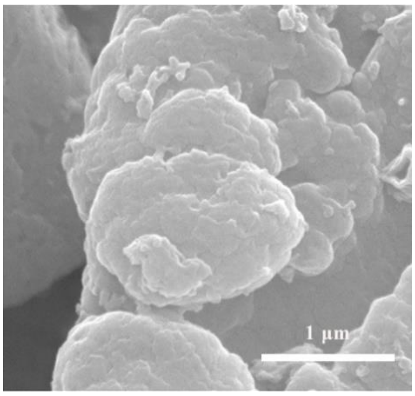

Figure S3. SEM image of CN.

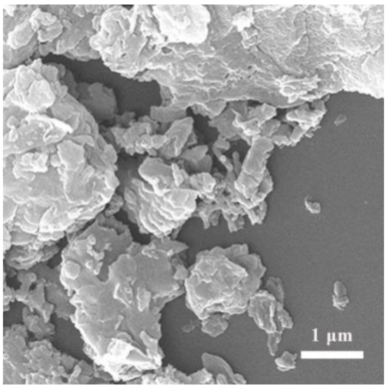

Figure S4. SEM image of CN-1.

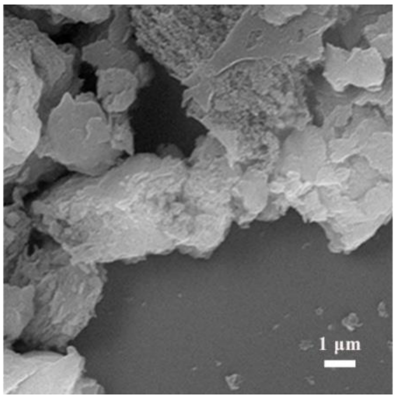

Figure S5. SEM image of CN-2.

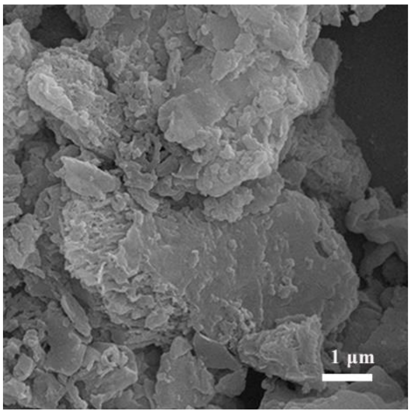

Figure S6. SEM image of CN-3.

Table S1. Elemental analysis of C and N content (wt.%) in CN, CN-1, CN-2 and CN-3.

Sample	N wt.%	C wt.%	C/N
CN	55.65	42.40	0.76
CN-1	53.59	43.32	0.81
CN-2	54.06	44.53	0.82
CN-3	53.81	44.47	0.83

Table S2. BET surface areaand H_2 production rateof CN and CN-2 samples.

Sample	BET surface area (m^2g^{-1})	H_2 production rate (μmol $h^{-1}g^{-1}$)
CN	5.26	101.2
CN-2	22.74	830

Table 3. Hydrogen evolution of CN-2 and comparison.with other reported g-C_3N_4 photocatalyst.

Sample	Light	H_2 evolution (μmol h^{-1})	Ref.
CN-2	λ >400 nm	830	This work
g-C_3N_4nanosheets	λ >420 nm	230	[1]
g-C_3N_4/C_{60}	λ >420 nm	266	[2]
PAN/ g-C_3N_4	λ >400 nm	370	[3]
S doped g-C_3N_4	λ >420 nm	525	[4]
C bridged g-C_3N_4	λ >420 nm	529	[5]
Cl doped g-C_3N_4	λ >420 nm	537	[6]

References

1. Xu, J.;Zhang,L. W. Shi,R.;Zhu, Y.F.Chemical exfoliation of graphitic carbon nitride for efficient heterogeneous photocatalysis.*J Mater Chem A* **2013**, *1*, 14766–14772.
2. Chen,X.;Chen,H.;Guan,L. J.;Zhen, J.M.;Sun, Z.J.;Du, P.W.;Lu, Y.L.;Yang, S.F. A facile mechanochemical route to covalently bonded graphitic carbon nitride (g-C3N4) and fullerene hybrid toward enhanced visible light photocatalytic hydrogen production. *Nanoscale* **2017**, *9*, 5615–5623.
3. He,F.;Chen,G.;Yu, Y.G.;Hao,S.;Zhou,Y. S.;Zheng,Y. Facile approach to synthesize g-PAN/g-C_3N_4 composites with enhanced photocatalytic H_2 evolution activity *Acs Appl Mater Inter* **2014**, *6*, 7171–7179.
4. Yang, C.; Zhang, S.S.;Huang, Y.; Lv, K.L.;Fang, S.; Wu, X.F.; Li,Q.; Fan, J.J. Sharply increasing the visible photoreactivity of g-C_3N_4 by breaking the intralayered hydrogen bonds. *Appl Surf Sci* **2020**, *505*, *144654*

5. Li, H.L.; Li, F.P.;Wang, Z.Y.;Jiao, Y.C.;Liu, Y.Y.;Wang, P.X.;Zhang, Y.;. Qin, X.Y.; Dai,Y.;Huang, B.B. Fabrication of carbon bridged g-C_3N_4 through supramolecular self-assembly for enhanced photocatalytic hydrogen evolution. *Appl Catal B-Environ* **2018**, *229*, 114–120.
6. Liu, C.Y.Zhang,Y. H.;Dong,F.; Reshak, A.H.;Ye, L.Q.;Pinna, N.;Zeng,C.;Zhang, T.R.;Huang, H.W. Chlorine intercalation in graphitic carbon nitride for efficient photocatalysis.*Appl Catal B-Environ* **2017**, *203*, 465–474.
